# Nodal Upstaging Evaluation After Robotic-Assisted Lobectomy for Early-Stage Non-small Cell Lung Cancer Compared to Video-Assisted Thoracic Surgery and Thoracotomy: A Retrospective Single Center Analysis

**DOI:** 10.3389/fsurg.2021.666158

**Published:** 2021-07-01

**Authors:** Filippo Tommaso Gallina, Enrico Melis, Daniele Forcella, Edoardo Mercadante, Daniele Marinelli, Serena Ceddia, Federico Cappuzzo, Sabrina Vari, Fabiana Letizia Cecere, Mauro Caterino, Antonello Vidiri, Paolo Visca, Simonetta Buglioni, Isabella Sperduti, Mirella Marino, Francesco Facciolo

**Affiliations:** ^1^Thoracic Surgery Unit, IRCCS Regina Elena National Cancer Institute, Rome, Italy; ^2^Medical Oncology 2, IRCCS Regina Elena National Cancer Institute, Rome, Italy; ^3^Medical Oncology 1, IRCCS Regina Elena National Cancer Institute, Rome, Italy; ^4^Radiology Unit, IRCCS Regina Elena National Cancer Institute, Rome, Italy; ^5^Department of Pathology, IRCCS Regina Elena National Cancer Institute, Rome, Italy; ^6^Department of Biostatistics, IRCCS Regina Elena National Cancer Institute, Rome, Italy

**Keywords:** NSCLC, robotic thoracic surgery (RATS), mediastinal lymphadenectomy, VATS, thoracic oncology

## Abstract

**Introduction:** The standard surgical procedures for patients with early-stage NSCLC is lobectomy-associated radical lymphadenectomy performed by using the thoracotomy approach. In the last few years, minimally invasive techniques have increasingly strengthened their role in lung cancer treatment, especially in the early stage of the disease. Although the lobectomy technique has been accepted, controversy still surrounds lymph node dissection. In our study, we analyze the rate of upstaging early non-small cell lung cancer patients who underwent radical surgical treatment using the robotic and the VATS techniques compared to the standard thoracotomy approach.

**Methods and Materials:** We retrospectively reviewed patients who underwent a lobectomy and radical lymphadenectomy at our Institute between 2010 and 2019. We selected 505 patients who met the inclusion criteria of the study: 237 patients underwent robotic surgery, 158 patients had thoracotomy, and 110 patients were treated with VATS. We analyzed the demographic features between the groups as well as the nodal upstaging rate after pathological examination, the number of dissected lymph nodes and the ratio of dissected lymph nodes to metastatic lymph nodes of the three groups.

**Results:** The patients of the three groups were homogenous with respect to age, sex, and histology. The postoperative major morbidity rate was significantly higher in the thoracotomy group, and hospital stay was significantly longer. The percentage of the mediastinal nodal upstaging rate and the number of dissected lymph nodes was significantly higher in the robotic group compared with the VATS group. The ratio of dissected lymph nodes to metastatic lymph nodes was significantly lower compared with the VATS group and the thoracotomy group.

**Discussion:** The prognostic impact of the R(un) status is still highly debated. A surgical approach that allows better results in terms of resection has still not been defined. Our results show that robotic surgery is a safe and feasible approach especially regarding the accuracy of mediastinal lymphadenectomy. These findings can lead to defining a more precise pathological stage of the disease and, if necessary, to more accurate postoperative treatment.

## Introduction

Lung cancer is the leading cause of cancer-related deaths worldwide. Non-small cell lung cancer (NSCLC) represents 80% of all lung cancers (1). Despite recent advances made in therapy, patients with NSCLC still present an estimated 5-year overall survival rate <25% for all stages (2). Current prognostic factors for this tumor include TNM staging, tumor size, and node positivity, as well as histological grade and histological subtypes; however, there is a need to improve the reliability of these with other indicators (3). Novel diagnostic surgical procedures improved the preoperative staging for patients with suspected NSCLC (4). Despite the accuracy of endoscopic procedures in finding pathological lymph nodes, a high number of postoperative pathological upstaging is still detected (5). In the last few decades, the surgical treatment of NSCLC has evolved toward an increasing use of minimally invasive techniques, at first with video-assisted thoracoscopic surgery (VATS) and more recently with robotics (6). Standard radical surgical treatment for early-stage NSCLC is lobectomy associated with radical lymphadenectomy (7, 8). Despite the fact that the open thoracotomy technique is considered the gold standard, minimally invasive lobectomy has been associated with improved perioperative and comparable long-term outcomes (9). However, controversy remains regarding lymph node assessment. Lymph node dissection is a crucial component in the surgical treatment of NSCLC. Survival in lung cancer after surgery depends on the number of pathological nodes (pN); thus, lymph node upstaging can be considered a surrogate for surgical quality of the procedure. Although previous studies have shown that VATS can yield an adequate lymphadenectomy, other studies have observed that nodal upstaging with VATS was significantly less common. A perceived benefit of robotic surgery is its ease of use for lymph node dissection. Thus, an accurate histopathological evaluation of the hilum-mediastinal lymph nodes seems to be more feasible. In this study, we evaluated patients with early-stage NSCLC who underwent pulmonary resection and radical lymphadenectomy with robotic technology compared to other surgical techniques. In 2012, we started a minimally invasive thoracic surgery program, first with the introduction of VATS and in 2016 with the launch of robotic surgery. We evaluated the nodal upstaging rate of VATS and robotic surgery compared with the gold standard, the thoracotomy approach. As a secondary aim, we retrospectively evaluated the value of the ratio of positive lymph nodes compared to removed lymph nodes.

## Methods and Materials

### Study Design

The study was designed as a single-center and retrospective case-matched analysis (VATS vs. thoracotomy vs. Robotic-Assisted Thoracic Surgery, RATS) in patients presenting with early stage NSCLC with clinical N0 who underwent curative surgery. Data for the analysis were retrieved from our lobectomy database. Up until 2012, our standard surgical technique for the treatment of early stage NSCLC was thoracotomy. From 2012, we started the VATS lobectomy program, first using the tri-portal or bi-portal approach and from 2014 the uni-portal technique. From 2016, we started the RATS lobectomy program using the Robotic Da Vinci technology, first with the Si model, then with the Xi model.

The general inclusion criteria for this study were patients diagnosed with NSCLC at stages I–II with clinical N0 disease undergoing anatomical lobectomy plus systematic lymph node dissection. The completeness of the lymphadenectomy has been evaluated in accordance with the IASLC definition regarding complete lymph node dissection of both N1 and N2 stations (10). Patients with clinical stages III–IV were excluded, and clinical N1 confirmed with endoscopic procedures, patients with SCLC, sublobar resections, and wedge resections were also excluded. Patients who had undergone preoperative chemotherapy or radiotherapy were excluded. Bi-lobectomy or pneumonectomy patients were not included in the study.

From January 2010 to December 2019, we performed a total of 1,352 lobectomies at our Institutes. In all, 505 patients were selected for our study in accordance with the inclusion criteria of this study. And 237 patients underwent robotic surgery, 158 patients underwent posterolateral thoracotomy, and 110 patients were treated with bi-portal or uni-portal VATS.

### Preoperative Staging

Preoperative investigations included thoracic and upper abdominal computed tomography (CT) and F18-fluorodeoxyglucose positron emission tomography (FDG-PET) used to establish the absence of multiple pulmonary lesions and the absence of hepatic, adrenal, or brain metastases, and also to evaluate hilar and mediastinal lymph node status. Bone scintigraphy was performed if clinically indicated (11). At the preoperative stage, lymph nodes were considered negative when the CT scan showed a short-axis < 1 cm and/or when the standardized uptake value was <3 in the PET scan, in accordance with the guidelines of nuclear medicine physicians. In the event of nodes > 1 cm and SUV in PET scan < 3, an endobronchial ultrasound transbronchial fine needle aspiration (EBUS-TBNA), an endoscopic ultrasound fine needle aspiration (EUS-FNA), mediastinoscopy, or VATS was performed to exclude malignancy (12). Before the operations were carried out, all patients had signed an informed consent to undergo a lobectomy. All patients who underwent VATS or RATS procedures were informed about the possibility of switching to thoracotomy in the case of unexpected technical problems during surgery. Before performing surgery, all patient cases were discussed in multidisciplinary meetings consisting of thoracic surgeons, oncologists, pathologists, radiotherapists, and pneumologists.

### Surgical Technique

All the procedures were performed by surgeons with demonstrated proof of experience with performing this technique. The posterolateral thoracotomy requires the patient to be positioned in the lateral decubitus. The incision started along the inframammary crease and extended below the tip of the scapula. It was then extended superiorly between the spine and the edge of the scapula, a short distance away. If necessary, the trapezius was also divided. The serratus anterior and latissimus dorsi muscles were identified and could be retracted. The intercostal muscles were then divided along the superior border of the ribs, and the thoracic cavity was accessed.

The VATS approach was performed with a 3-cm anterolateral non rib-spreading utility incision in the fifth intercostal space, splitting the serratus anterior along its muscle fibers. In the case of a bi-portal approach another additional 12-mm port, in the seventh intercostal space on the middle axillary line for using a 10-mm access, 30° camera was added (13).

Robotic surgery was performed by first using the Si da Vinci robot and after adopting the Xi version. The Si da Vinci robot is positioned at the head of the patient. The Xi da Vinci robot is positioned at the back of the patient. We always proceed performing a 3-cm utility incision at the 5th intercostal space anteriorly of the latissimus dorsi. The wound is usually protected with a soft tissue retractor. We then performed the other three operative ports under direct view guidance usually at the 8th or 9th intercostal space. We then started docking the robot. We always use a 30-degree stereoscopic robotic camera. Under direct view, the bed-assistant started to introduce the operative robotics arms.

The lobectomy technique, by thoracotomy, VATS, or RATS, was similar. The pulmonary vein, pulmonary artery, and lobar bronchus were individually isolated and divided with a vascular three-line stapler. A parenchymal stapler was also used for dividing incomplete fissures. In the VATS or robotic approach, the lobe was retrieved with an endoscopic bag.

The lymph node dissection was considered complete, when at least three mediastinal (N2) lymph node stations, always including station 7, were dissected, in addition to the intrapulmonary/hilar (N1) lymph nodes from station 10 and 11 (14).

### Histopathological Examination

All specimens were formalin fixed, paraffin-embedded, and stained with hematoxylin and eosin. Tumors were evaluated by an experienced pathologist and graded according to the World Health Organization classification for NSCLC (15). Pleural invasion, lymphatic involvement, and vascular involvement were determined by hematoxylin and eosin staining. In each histological examination the number of resected lymph nodes has been indicated.

### Statistical Analysis

Statistical analyses were performed using the Statistical Package for Social Sciences for Windows (SPSS® 23.0, Chicago, IL, USA). Non-parametric tests were used for comparisons, and data were expressed as the median (standard deviation). The significance threshold was *p* < 0.05.

## Results

In Table 1, all the demographic and surgical features were reported. The three groups were homogenous in terms of age, gender, tumor dimension, preoperative staging and histology. Median age was 66.5 ± 8.8 years. In total, 282 patients were male while 222 were female. All patients after preoperative staging were in stage I or II according to the 8th TNM classification without lymph node disease (cN0). No differences between groups were reported in terms of preoperative clinical stages (I, Ib, IIa, IIb). The three groups were homogeneous with respect to the type of lobectomies performed (*p* = 0.3).

**Table 1:** Demographic and surgical features in the Robotic, VATS, and Thoracotomy groups.

	**Open (*n* = 158)**	**VATS (*n* = 110)**	**RATS (*n* = 237)**	**All Patients (*n* = 505)**	***P*-value**
Age (years)	68.9 ± 7.8	64 ± 5.3	65.2 ± 6.3	66.5 ± 8.8	0.5
Gender (M/F)	94/64	63/47	134/103	282/222	0.4
Side (R/L)	91/67	73/37	113/124	228/274	0.4
Lobectomy (*n*, %)					0.3
RUL	50 (31.5)	38 (34.5)	55 (23.2)	142 (28.5)	
ML	2 (1.5)	10 (9.6)	14 (5.9)	26 (5.4)	
RLL	39 (24.8)	24 (21.8)	43 (18.2)	106 (21.0)	
LUL	35 (22.5)	22 (20.5)	77 (32.5)	134 (26.5)	
LLL	31 (19.7)	15 (13.6)	48 (20.2)	94 (18.6)	
T Dimension (mm)	2.9 ± 1.8	2.6 ± 1.0	2.7 ± 1.6	2.7 ± 1.2	0.2
Hospital stay (days)	7.12 ± 3.4	5.2 ± 1.2	5.7 ± 1.1	6.28 ± 1.9	0.04
Preoperative stage (*n*, %)					0.3
Ia	75 (47.5)	59 (55.5)	102 (43.0)	237 (46.9)	
Ib	43 (27.2)	29 (26.4)	73 (30.8)	146 (28.9)	
IIa	25 (15.8)	12 (10.8)	49 (20.7)	86 (17.1)	
IIb	15 (9.5)	8 (7.3)	13 (5.5)	36 (7.1)	
Post-operative complications (*n*, %)	23 (14.5)	6 (5.4)	13 (5.5)	63 (12.5)	0.03

Median hospital stay was 6.28 ± 1.9 days but the VATS and RATS groups showed to have shorter hospital stay compared with the open group. The postoperative major morbidity rate until 30 days after surgery was significantly higher in the thoracotomy group compared to the VATS group and the robotic group (23, 14.5% vs. 6, 5.4% vs. 13, 5.5%; *p* = 0.04). No deaths occurred during the 30 days.

**Figure 1:**
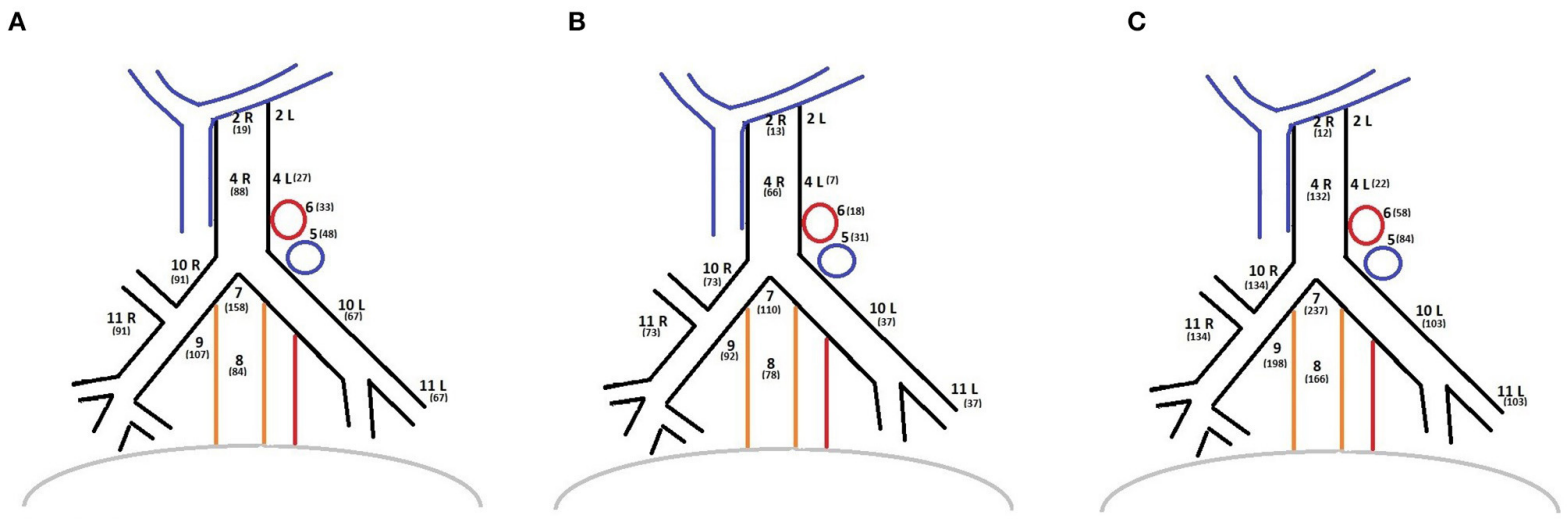
**(A)** Lymph nodes stations distribution of OPEN group. **(B)** Lymph nodes stations distribution of VATS group. **(C)** Lymph nodes stations distribution of ROBOTIC group.

The histopathological yield showed that in all groups the most frequent histological type was adenocarcinoma. The histological examination confirmed pN0 disease in 399 patients. A total of 48 patients presented with pN1 disease while 58 patients presented with pN2 (Table 2). The distribution of the dissected lymph node stations are reported in Figure 1. Stations 7, 10, and 11 were always dissected according to the IASLC guidelines. There were no differences between groups in terms of number of lymph node stations resected.

**Table 2:** Histopathological and staging features in the Robotic, VATS, and Thoracotomy groups.

	**Open (*n* = 158)**	**VATS (*n* = 110)**	**RATS (*n* = 237)**	**All Patients (*n* = 505)**	***P*-value**
Histology (*n*, %)					0.5
Adenocarcinoma	114 (72.1)	78 (70.9)	187 (78.9)	379 (75.0)	
Squamous cell carcinoma	23 (14.6)	19 (17.3)	36 (15.2)	78 (15.4)	
Neuroendocrine tumors	21 (13.3)	13 (11.8)	14 (5.9)	48 (9.6)	
pT (*n*, %)					0.3
1a	35 (22.2)	12 (10.9)	22 (9.3)	69 (13.7)	
1b	26 (16.5)	23 (20.9)	65 (27.4)	114 (22.6)	
1c	1 (0.6)	20 (18.2)	25 (10.5)	46 (9.1)	
2a	57 (36.1)	38 (34.5)	94 (39.7)	189 (37.4)	
2b	26 (16.5)	6 (5.5)	20 (8.4)	52 (10.3)	
3	12 (7.6)	9 (8.2)	11 (4.6)	22 (4.3)	
pN (*n*, %)					0.1
N0	118 (74.7)	95 (86.4)	187 (78.9)	399 (79.0)	
N1	15 (9.5)	8 (7.3)	24 (10.1)	48 (9.5)	
N2	25 (15.8)	7 (6.4)	26 (11.0)	58 (11.5)	

In Table 3, the nodal upstaging rate is reported. The percentage of nodal upstaging in patients from the three groups was similar, although in the robotic group, a higher percentage of patients compared to the VATS group presented an upstaging from pN0 to pN2, with a statistically significant difference (25, 15.9% vs. 26, 11.0% vs. 7, 6.4%; *p* = 0.04). No patient with neuroendocrine tumors (39 typical and 9 atypical carcinoids) presented a nodal upstaging after surgery in the three groups. No differences between groups were reported in case of an upstaging from pN0 to pN1.

**Table 3:** Nodal upstaging in the Robotic, VATS, and Thoracotomy groups.

	**Open (*n* = 158)**	**VATS (*n* = 110)**	**RATS (*n* = 237)**	**All patients (*n* = 505)**	***P*-value**
Nodal upstaging (%)	40 (25.3)	15 (13.6)	50 (21.1)	105 (20.8)	*p* = 0.1
Hilar upstaging (%)	15 (9.6)	8 (7.3)	24 (10.1)	47 (9.3)	*p* = 0.2
Mediastinal upstaging (%)	25 (15.9)	7 (6.4)	26 (11.0)	58 (11.5)	*p* = 0.04

The results of the number of dissected lymph nodes and the lymph nodes ratio are summarized in Table 4. The number of the total lymph nodes resected was significantly higher in the robotic and thoracotomy group than the VATS group (13, 3–41 vs. 15, 3–44 vs. 9, 3–25; *p* = 0.0001). Even though the number of hilar lymph nodes did not present any differences between groups, the number of resected lymph nodes in the mediastinal stations was significantly higher in the robotic group than the VATS group. The robotic surgery group showed a significantly lower value of lymph nodes ratio compared to the VATS (9.09, 4–67 vs. 18.55, 4–50; *p* = 0.0001) and the open surgery groups (9.09, 4–67 vs. 16.67, 2–100; *p* = 0.0001).

**Table 4:** Lymph nodes features in the Robotic, VATS, and Thoracotomy groups.

	**Open (*n* = 158)**	**VATS (*n* = 110)**	**RATS (*n* = 237)**	**All patients (*n* = 505)**	***P*-value**
Lymph nodes resected	13 ± 11.6	9 ± 5.7	15 ± 7.01	13 ± 8.2	0.0001
Hilar Lymph nodes resected	5 ± 3.1	3 ± 2.2	4 ± 2.0	4 ± 4.2	0.5
Mediastinal Lymph nodes resected	10 ± 8.2	7 ± 3.4	11 ± 9.6	12 ± 8.1	0.0001
Lymph nodes ratio (metastatic/resected)	16.7 ± 2.1	18.7 ± 1.7	9.1 ± 1.5	12.5 ± 2.1	0.001

## Discussion

The standard surgical treatment for early-stage NSCLC is lobectomy and radical hilum mediastinal lymphadenectomy (16). The concept of the resection status analysis in early-stage NSCLC is still under great debate where the “IASLC Lung Cancer Staging Project” analysis emphasizes the need for improving pathologic nodal staging. Our group has contributed to the new staging project (17) by evaluating the relevance of nodal dissection with respect to the R status. Survival of lung cancer after surgery depends on the number of pathological nodes; therefore, an adequate surgical lymph node dissection should be the first aim during surgery (18). Survival following surgery for node-negative non-small cell lung cancer is associated with the number of lymph nodes dissected and analyzed (19). Higher numbers of resected lymph nodes provide more complete staging and reduce the likelihood of missing metastatic lymph nodes (20). Although the preoperative staging technique has greatly improved, the number of metastatic lymph nodes that remain hidden is still significant. Therefore, lymph node upstaging after surgery could represent a quality indicator of treatment (21).

Up until two decades ago, the only surgical technique for treating NSCLC was thoracotomy but in the last few years minimally invasive techniques have significantly improved. The first minimally invasive technique adopted was video-assisted thoracoscopic surgery (VATS), demonstrating excellent morbidity and mortality outcomes (22). Short-term results of VATS compared with thoracotomy are well-documented: fewer complication rates, less postoperative pain, and shorter hospital stays (23). The long-term efficacy of VATS in comparison with the thoracotomy approach for lung cancer surgery is uncertain. In the last few years some authors showed the results of nodal upstaging in VATS procedures compared with thoracotomy, considered the gold standard (24). One of the most important comparative studies between VATS and open lobectomy and lymphadenectomy was performed by Licht et al., who evaluated the pathological results of 1,513 lobectomies for stage I NSCLC. The results showed that the VATS group reported a lower upstaging rate compared with the open group, but no differences in overall survival were found (25). On the other hand, Boffa et al. reported a similar nodal upstaging rate between the VATS and the open groups in a cohort of 11,500 patients from the Society of Thoracic Surgeons database (26). What the two studies have in common are the results concerning the number of mediastinal lymph nodes. VATS procedures showed a lower rate of resected lymph nodes, probably due to the more challenging dissection for a limited angle of maneuverability of thoracoscopic instruments.

The robotic approach represents a technological evolution of the VATS procedure (27). This leads to some technical advantages related to a better view of the operative field (3D instead of 2D), a simpler use of the instruments, more precise movements, and many possibilities deriving from the wide angle of maneuverability of the instruments, which is even superior to that of the human hand (28, 29). The first experience of upstaging analysis in patients with clinical stage I NSCLC, who underwent robotic segmentectomies or lobectomies, was reported by Wilson. In his multiple institutional study, upstaging was observed in 10.9% of cases, especially in those patients with larger lung tumors (30). In a single-center retrospective study, Zirafa et al. compared the upstaging rate of robotic lobectomy with the gold standard thoracotomy lobectomy showing a similar upstaging rate of robotic lobectomy but a higher upstaging rate evaluating the N2 disease in the robotic group (31). This result demonstrated that the robotic mediastinal lymph node dissection can be carried out safely, leading to better pathological staging of the disease.

Kneuertz et al., in a multicentric retrospective analysis that compared the three approaches (VATS, RATS, and open surgery), included the patients with clinical N0/N1 who had undergone lobectomy for NSCLC. Unlike in our study, the authors selected patients who had undergone radical or sampling lymphadenectomy. The overall rate of lymph node upstaging was highest with open lobectomy (21.8%), followed by robotic (16.2%), and VATS (12.3%) (*p* = 0.03) while no significant differences were seen in mediastinal N2 upstaging between groups (32).

In our study we compared minimally invasive techniques, such as robotic surgery and VATS, with open surgery. We evaluated the rate of nodal upstaging in a cohort of patients with cN0 disease who had undergone lobectomy and radical lymphadenectomy according to the IASLC definition with these three techniques. Then we evaluated the number of dissected lymph nodes and the ratio between metastatic lymph nodes and all the dissected lymph nodes. The results showed that robotic surgery can well replicate the dissection of the lymph nodes performed in open surgery. Nodal upstaging from No to N1 was similar in all the groups. The hilar lymph nodes (stations 10 and 11) should commonly be dissected in order to clear the view of the vascular or bronchial structures that must be closed to perform a lobectomy. Therefore, regardless of the surgical technique, an adequate number of hilar lymph nodes were always dissected. The nodal upstaging rate from N0 to N2 showed that the robotic surgery presented a significantly greater rate compared with the VATS approach. These results are probably due to the greater difficulties encountered in comfortably reaching all mediastinal areas with thoracoscopic instruments compared with the robotic technology. With robotic surgery, the three operative arms allow an excellent view of the anatomical limitations also of small surgical areas such as the mediastinal lymph node stations. The use of 3D imaging allows us to achieve better exposure of the anatomical structures that should be preserved during dissection. Station 4R can be accurately dissected discovering the trachea, preserving the vagus nerve, and respecting the limits of the superior vena cava and the azygos vein. Station 5 can be resected, avoiding the lesions of the laryngeal nerve. Station 7 can be explored by dissecting all the tissues up to the contralateral principal bronchus. We believe that by using robotic technology these steps can be performed safely and standardized more easily. Mediastinal lymph node dissection can cause bleeding and in cases of deep surgical sites, hemostasis can prove to be challenging. The accuracy of the robotic three arms allows a feasible search for the source of bleeding, and the use of bipolar forceps hemostasis can be easily carried out.

The analysis of the number of dissected lymph nodes and the lymph nodes ratio confirmed that robotic surgery enables us to perform a more accurate resection of all the mediastinal lymph node stations compared with the VATS approach. To obtain a more truthful pathological staging of the clinical early stage, non-small cell lung cancer allows setting a faster and more accurate postoperative oncological treatment in the event of positive lymph nodes. We believe that robotic surgery permits a more precise dissection than the VATS approach; therefore, patients who underwent a robotic lobectomy presented a higher rate of nodal upstaging.

This is one of the first studies to compare the three surgical techniques used for the treatment of early-stage NSCLC. Our study has some obvious limitations. Because of the retrospective and non-randomized nature of the analysis, it cannot be claimed with certainty that robotic surgery is the best approach compared with VATS for the mediastinal lymph nodes dissection. However, without prospective studies reported in the literature, we analyzed retrospectively our single center experience with the three approaches that showed a higher mediastinal nodal upstaging rate in the robotic group. The selection of the carcinoid tumors can be considered a limit of this retrospective study. Indeed, the PET-FDG has a poor sensitivity to detect the lymph nodes metastases and the nodal upstaging rate can be higher in comparison with the other histological types (33, 34). The patients with carcinoids presented preoperative contrast-enhanced CT scans without enlarged lymph nodes and after surgery no patient presented a nodal upstaging. Therefore, we think that the inclusion of these patients does not invalidate our results.

Other studies should be conducted to validate the oncological results of minimally invasive lymphadenectomy, but we believe that by using the robotic technique, a higher number of lymph nodes can be removed, and accurate pathological staging could bring better oncological outcomes. These findings place thoracic robotic surgery as a valid alternative to the open approach and support it as the gold standard for the surgical treatment for NSCLC.

## Data Availability Statement

The original contributions presented in the study are included in the article/supplementary material, further inquiries can be directed to the corresponding author/s.

## Ethics Statement

This study was carried out in accordance with the recommendations of our Ethics Committee. The protocol was reviewed and approved by the Comitato Etico Centrale IRCCS Lazio Found. Bietti. All subjects gave written informed consent in accordance with the Declaration of Helsinki.

## Author Contributions

FG designed the manuscript and drafted it. FG, EMel, DF, EMer, DM, SC, and FLC participated in the designing and drafting up of the manuscript. FC and FF critically revised it. PV, MM, SV, and SB coordinated the manuscript. All the authors contributed to the work during the years by their clinical or experimental activity, contributed to the article, and approved the submitted version.

## Conflict of Interest

The authors declare that the research was conducted in the absence of any commercial or financial relationships that could be construed as a potential conflict of interest.
